# Restoring of miR-193a-5p Sensitizes Breast Cancer Cells to Paclitaxel through P53 Pathway

**DOI:** 10.34172/apb.2020.071

**Published:** 2020-08-09

**Authors:** Monireh Khordadmehr, Roya Shahbazi, Behzad Baradaran, Sanam Sadreddini, Dariush Shanebandi, Khalil Hajiasgharzadeh

**Affiliations:** ^1^Department of Pathology, Faculty of Veterinary Medicine, University of Tabriz, 51665-1647, Tabriz, Iran.; ^2^Immunology Research Center, Tabriz University of Medical Sciences, 51666-14761, Tabriz, Iran.; ^3^Department of Immunology, Faculty of Medicine, Tabriz University of Medical Sciences, 51666-14761, Tabriz, Iran.

**Keywords:** Tumor-suppressor, Breast cancer, Proliferation, Migration, Gene expression

## Abstract

***Purpose:*** Recent evidence presented the important role of microRNAs in health and disease particularly in human cancers. Among those, miR-193 family contributes as a tumor suppressor in different benign and malignant cancers like breast cancer (BC) via interaction with specific targets. On the other hand, it was stated that miR-193 is able to modulate some targets in chemoresistant cancer cells. Therefore, the aim of this study was to evaluate the potential function of miR-193a-5p and paclitaxel in the apoptosis induction by targeting P53 in BC cells.

***Methods:*** At first, miR-193a-5p mimics were transfected to MDA-MB-231 BC cell line which indicated the lower expression level of miR-193a-5p. Subsequently, the transfected cells were treated with paclitaxel. Then, cell viability, apoptosis, and migration were evaluated by MTT, flow cytometry and DAPI staining, and scratch-wound motility assays, respectively. Moreover, the expression levels of P53 was evaluated by qRT-PCR.

***Results:*** The expression level of miR-193a-5p was restored in MDA-MB-231 cells which profoundly inhibited the proliferation (*P*<0.0001), induced apoptosis (*P* <0.0001) and harnessed migration (*P* <0.0001) in the BC cells and more effectiveness was observed in combination with paclitaxel. Interestingly, increased miR-193a-5p expression led to a reduction in P53 mRNA, offering that it can be a potential target of miR-193a.

***Conclusion:*** Taken together, it is concluded that the combination of miR-193a-5p restoration and paclitaxel could be potentially considered as an effective therapeutic strategy to get over chemoresistance during paclitaxel chemotherapy

## Introduction

Breast cancer (BC) is mentioned as the most commonly identified cancer in women worldwide. As more details, the American Cancer Society had predicted that there will be 266 120 and 2550 new incidences of BC in women and men, respectively. Also, survival rates for invasive BC are 90% and 83%, in 5 and 10 years, respectively.^1^ In BC treatment, surgery (including lumpectomy and mastectomy), radiotherapy (recommended for most patients having breast-conserving surgery), chemotherapy (before or after surgery), and hormone (anti-estrogen) therapy, and/or targeted therapy are considered as the main therapeutic strategies. However, these treatment approaches occasionally present some impediments like toxicity, relapse, different side effects and high-cost treatment. For these reasons, in recent years, a large number of researchers have interested and focused on novel therapy methods like microRNA replacement therapy.^2^

Convincing evidence propose miRNAs as one of the important regulators of gene expression with fundamental activities in main biological functions such as cell growth and proliferation, apoptosis, and migration.^3,4^ Emerging evidence proposes that miRNAs are frequently deregulated in different cancers and effect on biological processes through managing the pivotal tumor suppressor genes or oncogenes.^5^ Of note, these molecules have been contributed in cancer biology and development like cell growth and programmed cell death, angiogenesis, cell motility and migration, invasion and metastasis of various tumors like BC.^6,7^ In this regard, it was previously demonstrated that miR-193 family (including has-miR-193a and has-miR-193b) can prevent proliferation and cell cycle modulation in conventional cells. Indeed, it was clarified the tumor suppressor function for miR-193 in some tumors, and also demonstrated the important role of miR-193a in various cancers like bladder carcinoma, lung cancer, hepatocellular carcinoma, colon cancer, as well as BC.^8-14^ On the other hand, growing evidence propose that miR‐193 regulation can represent an innovative tool for the treatment of cancers. Besides, it was indicated that miR‐193a‐3p is able to decrease the expression level of some targets such as ING‐5, HOXC9, and PSEN‐1 in chemoresistant bladder cancer cells and the response to the chemotherapy could be improved.^15-17^ Our study demonstrated that miR-193a-5p could increase the sensitivity of BC cells to paclitaxel and also presented that miR-193a is involved in the management of apoptosis by targeting P53.

## Materials and Methods

### Cell culture and transfections

MDA-MB-231 cell line that originated from the human epithelial BC cells were acquired from the Pasture Institute, Tehran, Iran. Firstly, the cells were cultured in a standard cell culture condition based on our previous study.^18^ Subsequently, 2 × 10^5^ cells were seeded in a 6‐well plate. When the seeded cells reached to 50%-70% confluently, the cells were washed with PBS (phosphate‐buffered saline) and the miR‐193a-5p mimic transfection was performed using JetPEI reagent (Polyplus‐transfection, Strasbourg, France). To find the appropriate dosage of transfected miRNA and optimal time, pilot assessments were carried out^19^ in three 24, 48, 72 hours times intervals, and three dosages of 5, 7.5, 10 nmol to obtain the effective time and dosage, respectively. MiRNA from *Caenorhabditis elegans* (MISSION2® miRNA, Sigma-Aldrich Co.), with no sequence homology to the human gene, was used for transfection of negative controls (Table 1).

**Table 1:** Primer sequences used in the present study

**Primer name**	**Forward/ Reverse**	**Sequences**
P53	F R	5'-TCTTCCTGCCCACCATCTACTC-3' 5'-TGCAGCCTGTACTTGTCCGTC-3'
β–actin	F R	5´- TCCCTGGAGAAGAGCTACG -3´ 5´- GTAGTTTCGTGGATGCCACA -3´
U6 snRNA	Target sequence	5´-GUGCUCGUUCGGCAGCACACAUAUACUAAAAUUGGAACGAUACAGAGAG AAGAUUAGCAUGGCCCCUGCGCAAGGAUGACACGCAAAUUCGUGAAGCGU UCCAUAUUUUU-3´
Has-miR-193a-5p	Target sequence	5'-UCA UCU CGC CCG CAA AGA CCC A-3'
C. elegans miRNA	Target sequence	5'-CGGUACGAUCGCGGCGGGAUAUC-3'

### MTT assay for cell viability

The MTT (3‐ (4, 5‐dimethylthiazol‐2‐yl) ‐2,5‐diphenyltetrazolium bromide) assay was used to evaluate the survival of the cells. Indeed, this assay was exerted to determine the value of 50% of inhibition (IC50) for miR-193a-5p and paclitaxel. After assessment of the IC50 values for miR-193a-5p and paclitaxel, the cells transfected with miR-193a-5p and treated with paclitaxel. Briefly, 2 × 10^3^ of miR‐193a-5p transfected MDA-MD-231 cells and the negative control cells were separately seeded into 96‐well plates. Subsequently, the transfected cells were treated with different doses of paclitaxel ranging from 0.15 to 5 µg/ml (Mylan, Saint-Priest, France) after 24 hours. MTT solution^18^ was utilized to incubate the cells in a cell incubator at 37℃ for 4 hours. Then, the cell culture medium was depleted from each well. And 200 μL of DMSO (dimethyl sulfoxide) plus 25 μL of Sorenson’s buffer were added to each well for solubilization of MTT formazan crystals and incubated for 30 minutes at 37°C. Finally, the absorbance of each well was evaluated by using a microplate reader (Sunrise, Tecan, Switzerland) at 490–570 nm.

### RNA extraction, RT-PCR, and qRT‐PCR for miRNA and genes expression

Total RNA was isolated from the cell pellets (approximately 1 × 10^6^ cell) using RiboEx reagent (Gene All Biotechnology, Seoul, South Korea) and the quality and concentration of RNA was determined by Nano-Drop spectrophotometer (Thermo Fisher Scientific Life Sciences, USA). Moreover, the integrity of the RNA was checked by agarose gel electrophoresis. For miR‐193a-5p (cDNA) synthesis, the miRNA Reverse Transcription Kit (Exiqon, Vedbaek, Denmark) was used according to the manufacturer’s protocol. In parallel, cDNA synthesis for the P53 gene was performed using first strand cDNA synthesis kit (Thermo, USA) according to the suggested protocol. A Bio‐Rad thermal cycler (Bio‐Rad Laboratories, Inc., Hercules, CA) was utilized for applying temperatures.

Quantification of miRNA and mRNA expression levels was performed using a standard SYBR Green PCR pre-mix (Ampliqon, Odense, Denmark) in a Light-Cycler 96 System (Roche Diagnostics GmbH, Mannheim, Germany). Reaction tubes comprised 5 µL of 2X SYBR green premix, 0.25 µL of 4 pmol/μl primers and 0.5 µL of relating cDNA and nuclease-free water (up to 10 µL). The cycling carried out by 94°C for 10 seconds, 59°C for 30 seconds and 72°C for 20 seconds. Finally, the Ct values were analyzed by the 2^−ΔΔCT^ method. Of note, all reactions were considered in triplicates. β-actin was used as a housekeeping gene for mRNA expression analysis, while U6 was employed for miR-193a-5p normalization. The sequences of primers for P53 are provided in Table 1. Primer sets were purchased from Bioneer, Korea.

### Flow cytometry assay and DAPI staining for apoptosis evaluation

Annexin V/propidium iodide assay was used to assess apoptosis analysis. In this way, there were four experiment groups comprising control, miR-193a-5p, paclitaxel, and combined miR-193a-5p/paclitaxel. The cells were seeded at a density of 2 × 10^5^ cells in the 6-well plates for 24 hours at 37°C, and the treatments were performed. Twenty-four hours later, the cultured cells washed with PBS and trypsinized by Trypsin/EDTA 2.5%. Then 3 mL complete medium was added to the cells and carried over the 1.5 mL micro-tubes. Then, those were centrifuged at 1200 rpm for 5 minutes, and the supernatant was aspirated. Eventually, the staining was performed using Annexin V-FITC and Propidium Iodide Double Staining Kit (Invitrogen, USA) according to the manufacturer’s instruction. The analysis was performed using FlowJo software on the BD flow cytometry.

Moreover, DAPI (4´6-Diamidino-2-phenylindole) staining was applied to apoptosis evaluation. For this purpose, 1.5 × 10^4^ of BC cells were seeded into 96-well plates. Here, four experiment groups including control, miR-193a-5p, paclitaxel, and combined miR-193a-5p/paclitaxel were considered. After passing the incubation time for 24 hours at 37°C, the cells washed by PBS and then fixed with 4% paraformaldehyde for 20 minutes. After that, the cells were washed with PBS, 0.3% Triton X-100 was used to permeabilization for 10 minutes, and the cells were incubated for 10 minutes by 10 µg/mL DAPI. Eventually, the cells were washed with PBS and evaluated by a Cytation Five Cell Imaging Multi-Mode Reader (BioTek, VT).

### Wound healing assay for migration cells

The wound healing (scratch) assay was conducted for cell migration examination. In this way, we firstly seeded 10 × 10^4^ MDA-MB-231 cells into 24‐well plates. After reaching a confluence of 70%, the treatments were conducted in four experiment groups including control, miR-193a-5p, paclitaxel, and combined miR-193a-5p/paclitaxel. Twenty-four hours later, a gap was created using sterile sampler tip on the cell surface and the detached cells were washed using PBS. Migration of the cells to the “wound area” was observed and documented at 0, 24, and 48 hours by an inverted microscope (Optika Microscopes, Bergamo, Italy) and the number of the migrated cells were computed from multiple microscopic areas.

### Statistical analysis

For statistical analysis of the present data, we used GraphPad Prism software (GraphPad Prism 4.0, San Diego, CA). The statistical significance differences between the experimental groups were evaluated by Student’s *t* and ANOVA tests and a *P* < 0.05 was considered statistically significant.

## Results

### The expression level of miR-193a-5p enhanced in BC cells following the miR-193a-5p mimic transfection

**Figure 1:**
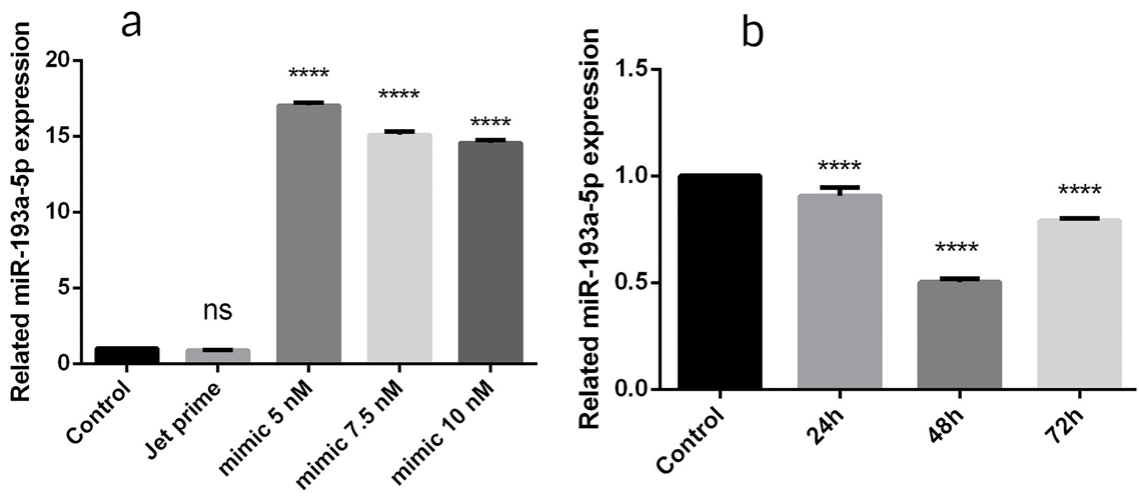
To find the appropriate dosage of transfected miRNA and assessment time, three dosages of 5, 7.5, and 10 nmol (a) and three times intervals of 24, 48, 72 h (b) were assessed. The results showed that 5 nmol in the 24 h was the most effective dosage in the most effective time (*P* < 0.0001). The data represent mean ± SD (n = 3), **** *P* < 0.0001.

To find the effective time and dosage, three designated times (24, 48 and 72 hours) and three miRNA doses (5, 7.5 and 10 nmol) were evaluated. According to the findings, 5 nmol of miRNA in 24 hours was found to be the most effective adjustment (*P*  < 0.0001) (Figure 1) and this setting was selected for the rest of examinations. In the current study, qRT-PCR indicated that the miR-193a-5p mimic transfection resulted in the efficient restoration of miR-193a-5p in BC cells.

### The cell viability substantially decreased through the combination of miR-193a-5p/paclitaxel in BC cells 

**Figure 2:**
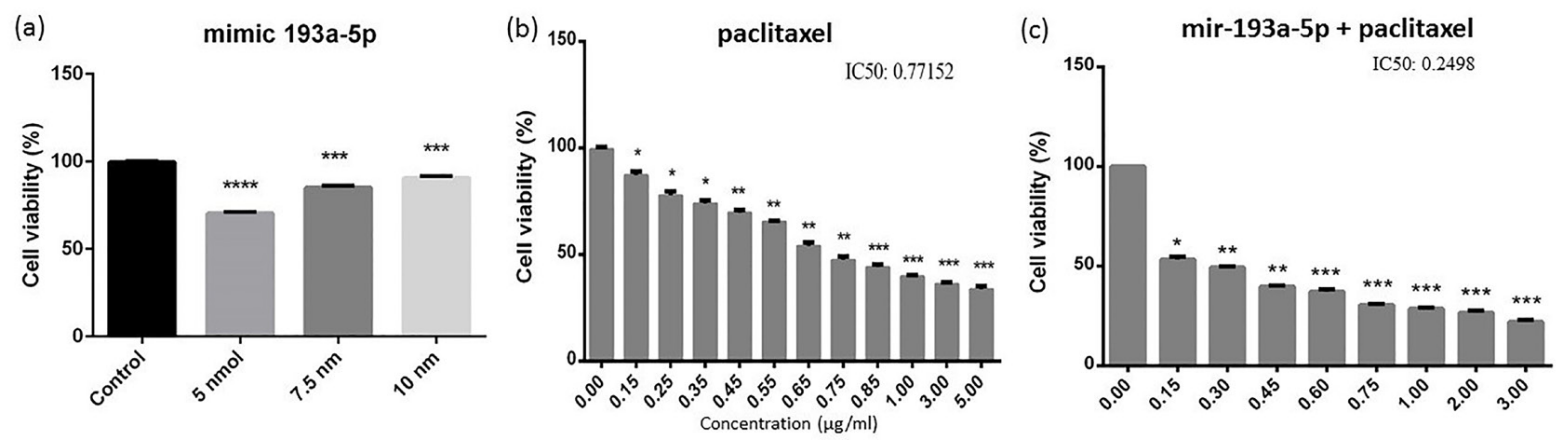
Effect of miR-193a-5p/paclitaxel on breast cancer MDA-MB-231 cells viability. The cells were adjusted with a different doses of miR-193a-5p, paclitaxel, and combination of miR-193a-5p/paclitaxel. Then, cell viability was evaluated by MTT assay. The data represent mean ± SD (n = 3). * *P* < 0.05; ** *P* < 0.01; *** *P* < 0.001; **** *P* < 0.0001.

To demonstrate the effects of miR-193a-5p and paclitaxel on the cell growth or apoptosis in MDA-MB-231 BC cell line, the MTT assay was conducted to determine the IC50 value of paclitaxel (IC50 = 0.77152). Interestingly, here it was shown that the replacement of miR-193a-5p in combination with paclitaxel led to a remarkable difference in cell proliferation. In more details, the present results exhibited that the restoration of miR-193a-5p could diminish the IC50 rate of paclitaxel (IC50 = 0.2498) in BC cells (Figure 2). Thus, it was suggested that contemporary restoration of miR-193a-5p and application of paclitaxel is able to reduce the cell proliferation in BC cells *in vitro* .

### The expression level of P53 significantly altered through the combination of miR-193a-5p/paclitaxel in BC cells

**Figure 3:**
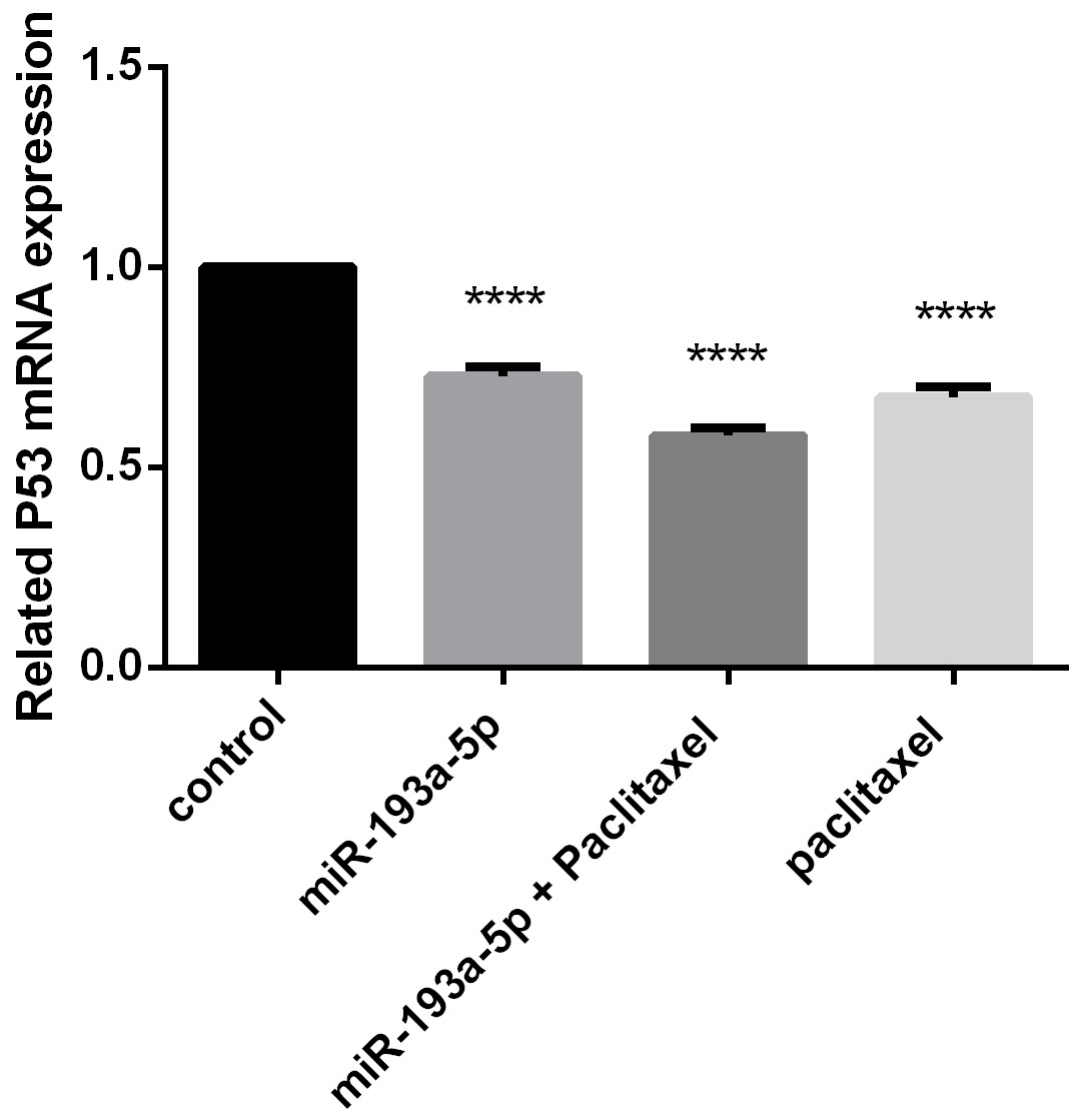
The effect of miR-193a-5p replacement and paclitaxel on P53 expression levels in breast cancer MDA-MB-231 cells by Q-RT-PCR assay. The data represent mean ± SD (n = 3). **** *P* < 0.0001.

To indicate the expression level of P53 after transfection of miR-193a-5p mimic in MDA-MB-231 BC cells, qRT-PCR was used which demonstrated a considerable decrement in the level of P53 compared to the control group (*P* < 0.0001) (Figure 3). Of considerable interest, there was a marked difference between miR-193a-5p restoration and combined miR-193a-5p/paclitaxel groups (*P* < 0.0001). Therefore, it was proposed that miR-193a-5p replacement/paclitaxel could induce apoptosis in BC cells *in vitro* .

### Restoration of miR-193a-5p in combination with paclitaxel could sensitize the BC cells to apoptosis

**Figure 4:**
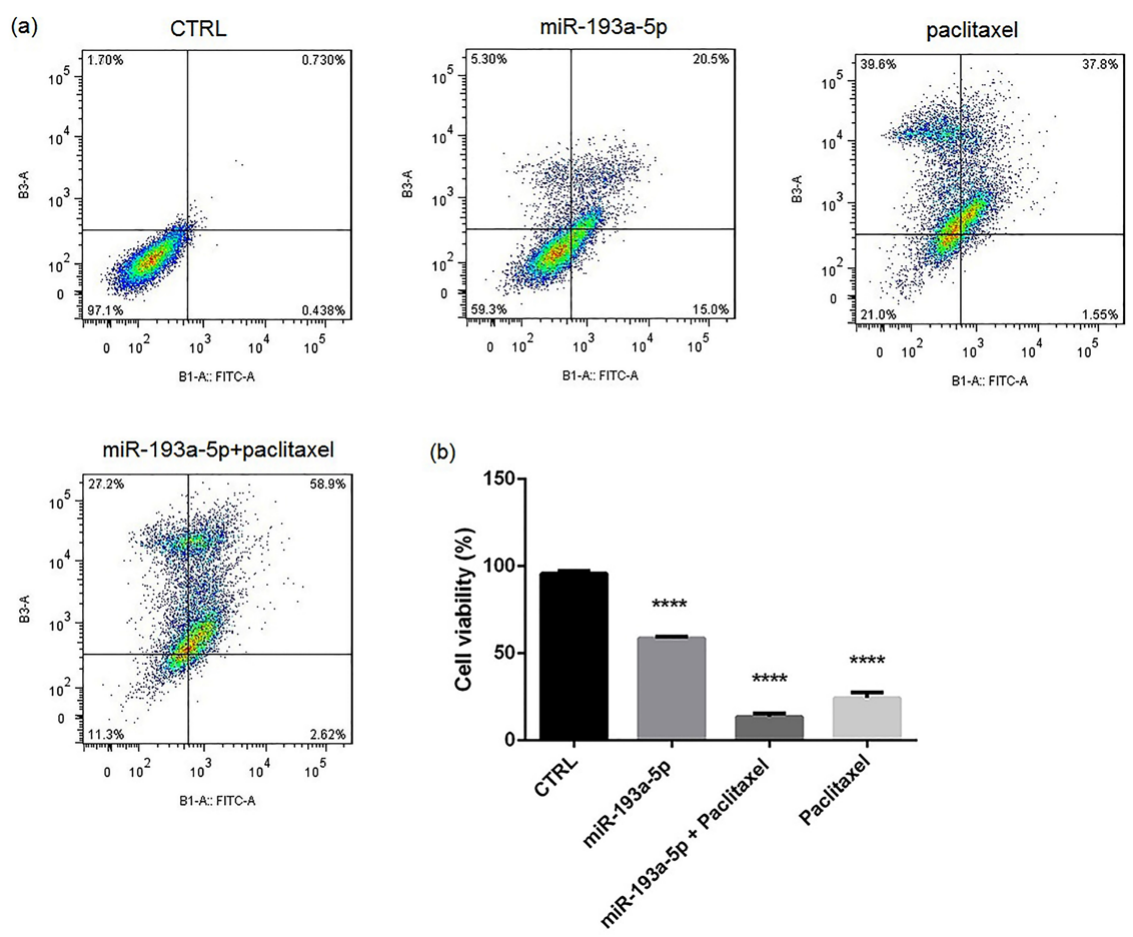
The effect of miR-193a-5p restoration and paclitaxel on apoptosis induction in breast cancer MDA-MB-231 cells by flow cytometry assay (Annexin V/PI staining) which significantly decreased cell viability. The data represent mean ± SD (n = 3). **** *P* < 0.0001.

**Figure 5:**
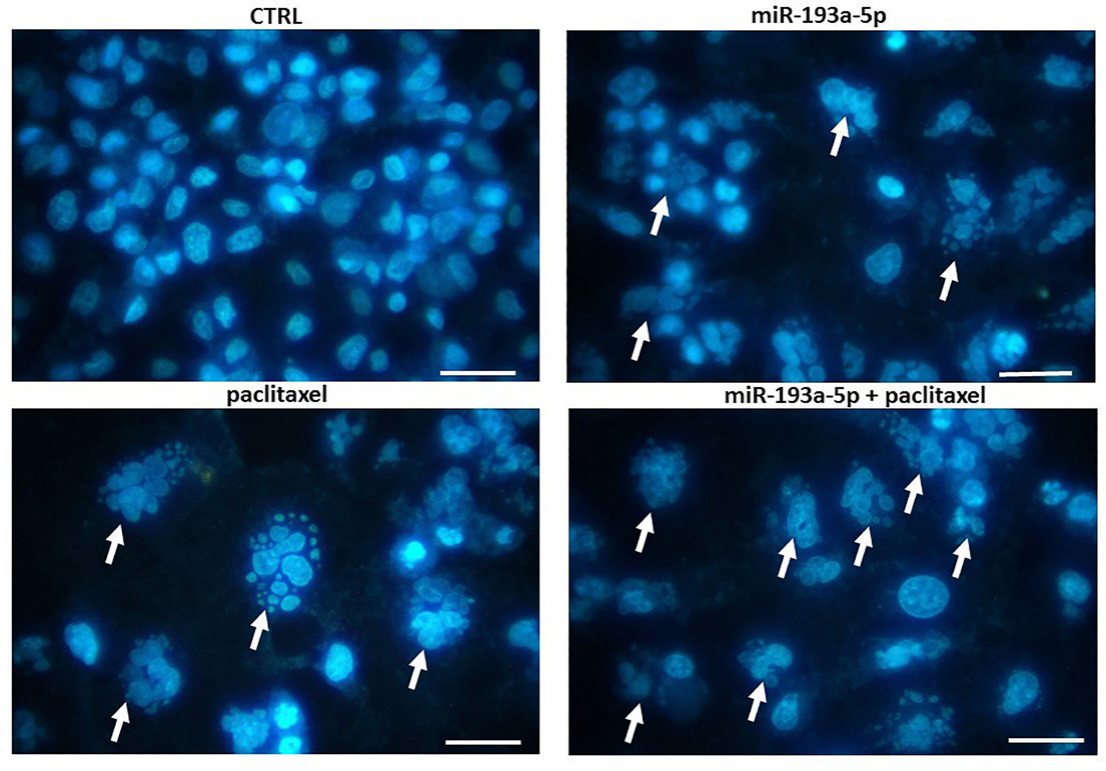
DAPI staining for evaluation of apoptosis. MiR-193a-5p restoration increased the apoptosis rate in breast cancer MDA-MB-231 cells particularly to gather with paclitaxel. Arrows demonstrate apoptotic cells in different groups. Scale bar = 200 µm.

Annexin V/PI and then DAPI staining assays were used to comprehend the impact of miR-193a-5p, paclitaxel and combined miR-193a-5p/paclitaxel on apoptosis. The flow cytometry test revealed clear differences in apoptosis rates after replacement of miR-193a-5p (Figure 4), particularly in the combination with paclitaxel (*P* < 0.0001). In the same line, the results of DAPI staining (Figure 5) approved the flow cytometry and pointed the significant difference in apoptosis rate in the combination of miR-193a-5p/paclitaxel when compared with other groups (*P* < 0.0001).

### Combination of miR-193a-5p/paclitaxel notably inhibited the migration ability of BC cells

**Figure 6:**
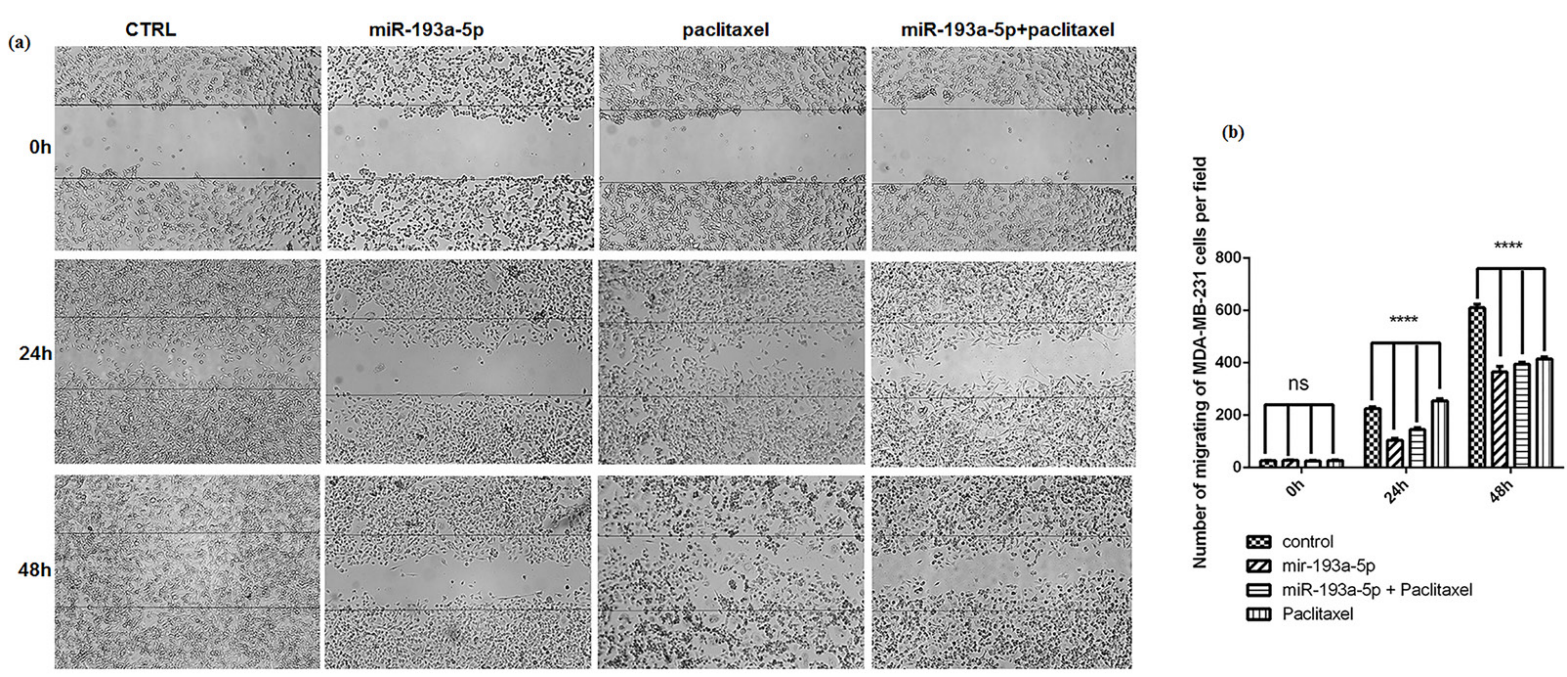
The effect of miR-193a-5p replacement and paclitaxel on cell migration in breast cancer MDA-MB-231 cells by wound healing assay. The quantity of the migrated cells into the wound area was evaluated after 24 and 48 h. The replaced miRNA significantly decreased cell migration, especially in combination with paclitaxel. The data represent mean ± SD (n = 3), **** *P* < 0.0001.

As previously described, the scratch test was performed to investigate the effect of miR-193a-5p and paclitaxel on the motility and migration ability of BC cells. These findings represented that miR-193a-5p and paclitaxel could strongly repress the migration ability of MDA-MB-231 cells (*P* < 0.0001) (Figure 6), particularly when the combination of miR-193a-5p/paclitaxel was used.

## Discussion

Despite extensive research on cancer in recent years, finding an effective cancer treatment remains a concern in this subject. Concerning the considerable effects of miRNAs in the modulation of essential cell functions, those enable forcefully to effect on drug resistance mechanisms by repression of special targets especially in chemotherapy management, which is considered as the most effective cancer therapeutic strategy for various common cancers.^7,20^ In this way, paclitaxel is an extensively administrated chemotherapeutic agent, and although, it is proposed as a highly effective agent in the treatment of BC, a significant percentage of treated patients show resistance to paclitaxel therapy.^21^

In the present work, the concurrent effect of miR-193a-5p replacement with paclitaxel was evaluated. These findings revealed the tumor-suppressive function of miR-193a-5p in BC cells by targeting P53. The MTT assay and DAPI staining data strongly clarified that together administration of miR-193a-5p/paclitaxel could be more effective in the promotion of apoptosis and also inhibition of proliferation. Therefore, the present results suggested that miR-193a-5p enable to improve the efficacy of paclitaxel in apoptosis induction through the P53 pathway which is mainly involved in programmed cell death. In addition, the qRT-PCR assay demonstrated a significantly lower expression level of P53 which can strongly approve the results of MTT assay and DAPI staining.

Many of the involved miRNAs in BC such as miR-18a (as an oncomiR), miR-34a and miR-143 (as tumor suppressor miRNAs) manage cancer cell growth and proliferation by modulating P53 expression and related apoptotic pathways.^22-24^

Previously, it was indicated that diminished function of miR‐ 193a‐3p generated by the sponge impact of Linc00152 was related to oxaliplatin resistance in colon cancer cells through the AKT signaling pathway.^25^ Likewise, it was understood that the miR‐193b overexpression in BC cells can raise sensitivity to doxorubicin by Mcl‐1.^26^ Also, convincing evidence indicated that miR‐193b clearly augmented the anticancer result of cisplatin by targeting of Mcl‐1 in liver cancer.^27^ Another researchers reported that miR-203 overexpression can sensitize paclitaxel-resistant colorectal cancer cells to chemotherapy through SIK2 targeting *in vitro* .^28^ In this connection, very recently, it was proposed that combination of miR-200c restoration with cisplatin is able to induce apoptosis and inhibit migration in gastric cancer cells.^29^ Therefore, the present and recent related data augment the important role of miRNAs in the chemoresistance of cancer cells which may open a new insight in the treatment of cancers.

On the other hand, here, results of the scratch test indicated a remarkable reduction in migration ability of BC cells subsequent restoration of miR-193a-5p/paclitaxel. Thus, it was concluded that combined miR-193a-5p/paclitaxel can sensitize BC cells to chemotherapy. Accordingly, increased resistance to carboplatin has been reported after upregulation of miR‐193b by directly targeting CRIM1 and IFIT2 in ovarian cancer cells.^30^

In conclusion, the present results demonstrated the important role of miR-193a-5p in the modulation of BC cell proliferation, migration and apoptosis. Taken together, it was concluded that miR-193a-5p could improve the sensitivity of BC cells to paclitaxel through targeting P53, and the combined cancer therapy insights can enable the improvement of a therapeutic to affect chemotherapy response.

## Ethical Issues

Not applicable.

## Conflict of Interest

Authors declare no conflict of interest in this study.

## Acknowledgments

The authors are grateful to the Faculty of Veterinary Medicine, University of Tabriz, Tabriz, Iran and also the Immunology Research Center, Tabriz University of Medical Sciences, Tabriz, Iran for the financial support.

## References

[1] American Cancer Society. Cancer Facts & Figures. Atlanta: American Cancer Society; 2018.

[2] Tang GH, Tang M, Xie YJ (2013). The role of miRNAs in gastric cancer. J Gastroint Dig Syst.

[3] van Schooneveld E, Wildiers H, Vergote I, Vermeulen PB, Dirix LY, Van Laere SJ (2015). Dysregulation of microRNAs in breast cancer and their potential role as prognostic and predictive biomarkers in patient management. Breast Cancer Res.

[4] Rothschild SI (2014). microRNA therapies in cancer. Mol Cell Ther.

[5] Minami A, Shimono Y, Mizutani K, Nobutani K, Momose K, Azuma T (2013). Reduction of the ST6 β-galactosamide α-2,6-sialyltransferase 1 (ST6GAL1)-catalyzed sialylation of nectin-like molecule 2/cell adhesion molecule 1 and enhancement of ErbB2/ErbB3 signaling by microRNA-199a. J Biol Chem.

[6] Goldberger N, Walker RC, Kim CH, Winter S, Hunter KW (2013). Inherited variation in miR-290 expression suppresses breast cancer progression by targeting the metastasis susceptibility gene Arid4b. Cancer Res.

[7] Khordadmehr M, Shahbazi R, Ezzati H, Jigari-Asl F, Sadreddini S, Baradaran B (2019). Key microRNAs in the biology of breast cancer; emerging evidence in the last decade. J Cell Physiol.

[8] Grossi I, Salvi A, Abeni E, Marchina E, De Petro G (2017). Biological function of microRNA193a-3p in health and disease. Int J Genomics.

[9] Lv L, Deng H, Li Y, Zhang C, Liu X, Liu Q (2014). The DNA methylation-regulated miR-193a-3p dictates the multi-chemoresistance of bladder cancer via repression of SRSF2/PLAU/HIC2 expression. Cell Death Dis.

[10] Wang J, Yang B, Han L, Li X, Tao H, Zhang S (2013). Demethylation of miR-9-3 and miR-193a genes suppresses proliferation and promotes apoptosis in non-small cell lung cancer cell lines. Cell Physiol Biochem.

[11] Heller G, Weinzierl M, Noll C, Babinsky V, Ziegler B, Altenberger C (2012). Genome-wide miRNA expression profiling identifies miR-9-3 and miR-193a as targets for DNA methylation in non-small cell lung cancers. Clin Cancer Res.

[12] Salvi A, Conde I, Abeni E, Arici B, Grossi I, Specchia C (2013). Effects of miR-193a and sorafenib on hepatocellular carcinoma cells. Mol Cancer.

[13] Teng Y, Ren Y, Hu X, Mu J, Samykutty A, Zhuang X (2017). MVP-mediated exosomal sorting of miR-193a promotes colon cancer progression. Nat Commun.

[14] Noh H, Hong S, Dong Z, Pan ZK, Jing Q, Huang S (2011). Impaired microRNA processing facilitates breast cancer cell invasion by upregulating urokinase-type plasminogen activator expression. Genes Cancer.

[15] Li Y, Deng H, Lv L, Zhang C, Qian L, Xiao J (2015). The miR-193a-3p-regulated ING5 gene activates the DNA damage response pathway and inhibits multi-chemoresistance in bladder cancer. Oncotarget.

[16] Lv L, Li Y, Deng H, Zhang C, Pu Y, Qian L (2015). MiR-193a-3p promotes the multi-chemoresistance of bladder cancer by targeting the HOXC9 gene. Cancer Lett.

[17] Deng H, Lv L, Li Y, Zhang C, Meng F, Pu Y (2015). The miR-193a-3p regulated PSEN1 gene suppresses the multi-chemoresistance of bladder cancer. Biochim Biophys Acta.

[18] Ghazanchaei A, Mansoori B, Mohammadi A, Biglari A, Baradaran B (2018). Restoration of miR-152 expression suppresses cell proliferation, survival, and migration through inhibition of AKT-ERK pathway in colorectal cancer. J Cell Physiol.

[19] Aletaha M, Mansoori B, Mohammadi A, Fazeli M, Baradaran B (2017). Therapeutic effects of bach1 siRNA on human breast adenocarcinoma cell line. Biomed Pharmacother.

[20] Weidhaas JB, Babar I, Nallur SM, Trang P, Roush S, Boehm M (2007). MicroRNAs as potential agents to alter resistance to cytotoxic anticancer therapy. Cancer Res.

[21] Zasadil LM, Andersen KA, Yeum D, Rocque GB, Wilke LG, Tevaarwerk AJ (2014). Cytotoxicity of paclitaxel in breast cancer is due to chromosome missegregation on multipolar spindles. Sci Transl Med.

[22] Song L, Lin C, Wu Z, Gong H, Zeng Y, Wu J (2011). miR-18a impairs DNA damage response through downregulation of ataxia telangiectasia mutated (ATM) kinase. PLoS One.

[23] Mansoori B, Mohammadi A, Shirjang S, Baghbani E, Baradaran B (2016). Micro RNA 34a and let-7a expression in human breast cancers is associated with apoptotic expression genes. Asian Pac J Cancer Prev.

[24] Ng EK, Li R, Shin VY, Siu JM, Ma ES, Kwong A (2014). MicroRNA-143 is downregulated in breast cancer and regulates DNA methyltransferases 3A in breast cancer cells. Tumour Biol.

[25] Yue B, Cai D, Liu C, Fang C, Yan D (2016). Linc00152 functions as a competing endogenous RNA to confer oxaliplatin resistance and holds prognostic values in colon cancer. Mol Ther.

[26] Long J, Ji Z, Jiang K, Wang Z, Meng G (2015). miR-193b modulates resistance to doxorubicin in human breast cancer cells by downregulating MCL-1. Biomed Res Int.

[27] Yin W, Nie Y, Zhang Z, Xie L, He X (2015). miR-193b acts as a cisplatin sensitizer via the caspase-3-dependent pathway in HCC chemotherapy. Oncol Rep.

[28] Liu Y, Gao S, Chen X, Liu M, Mao C, Fang X (2016). Overexpression of miR-203 sensitizes paclitaxel (Taxol)-resistant colorectal cancer cells through targeting the salt-inducible kinase 2 (SIK2). Tumour Biol.

[29] Ghasabi M, Majidi J, Mansoori B, Mohammadi A, Shomali N, Shirafkan N (2019). The effect of combined miR-200c replacement and cisplatin on apoptosis induction and inhibition of gastric cancer cell line migration. J Cell Physiol.

[30] Ziliak D, Gamazon ER, Lacroix B, Kyung Im H, Wen Y, Huang RS (2012). Genetic variation that predicts platinum sensitivity reveals the role of miR-193b* in chemotherapeutic susceptibility. Mol Cancer Ther.

